# *Plasmodium* in the bone marrow: case series from a hospital in Pakistan, 2007–2015

**DOI:** 10.1186/s12936-021-03792-1

**Published:** 2021-06-08

**Authors:** Muhammad Shariq Shaikh, Basim Ali, Mahin Janjua, Ayesha Akbar, Syed Arish Haider, Bushra Moiz, Ahmed Raheem, John Kevin Baird, Mohammad Asim Beg

**Affiliations:** 1 Department of Pathology and Laboratory Medicine, The Aga Khan University Hospital, Stadium Road, 74800 Karachi, Pakistan; 2 Aga Khan University, Karachi, Pakistan; 3 Eijkman-Oxford Clinical Research Unit, Jakarta, Indonesia; 4 Centre for Tropical Medicine and Global Health, Nuffield Department of Medicine, University of Oxford, Oxford, UK

**Keywords:** Malaria, Pakistan, Reticuloendothelial, Bone marrow, *Plasmodium*

## Abstract

**Background:**

Malaria is a life-threatening, multisystem disease caused by the plasmodial parasite with a global incidence of approximately 229 million annually. The parasites are known to have unique and crucial interactions with various body tissues during its life cycle, notably the liver, spleen, and recent work has shown the bone marrow to be a reservoir of infection.

**Methods:**

This study is a case series of patients in whom examination of bone marrow revealed malarial parasites. A retrospective record review of 35 parasite-positive bone marrow specimens examined at Aga Khan University Hospital (AKUH), Karachi, Pakistan, over the years 2007 to 2015 was conducted. Bone marrow aspirates were collected as per International Council for Standardization in Haematology (ICSH) guidelines.

**Results:**

The median age of patients was 22 years (range 1–75), and 60 % (n = 21) were male. 22 patients had evidence of *Plasmodium falciparum*, 12 had evidence of *Plasmodium vivax* and 1 patient had a mixed infection. Gametocytes and trophozoites were the most common stages identified on both peripheral blood and bone marrow examinations. Indications for bone marrow examination included fever of unknown origin and the workup of cytopenias and malignancies.

**Conclusions:**

The incidental finding of *Plasmodium* in samples of bone marrow suggests the reticuloendothelial system may be regularly harbour these parasites, be the infection acute or chronic in character.

## Background

Acute malaria is a life-threatening, multisystem disease caused by protozoan of the Genus *Plasmodium* that infect hundreds of millions of people annually, with several hundred thousand not surviving [1]. These parasites occur as different forms in various tissues, including liver, blood, spleen, and bone marrow [2]. *Plasmodium vivax*, in particular, exhibits specific molecular traits that would appear to accord with strong tropisms for haemopoietic tissues [3–5]. Merozoites of that species require transferrin receptor 1 (CD71) to invade erythrocytes [6], a molecule that vanishes from the surface of that cell during early reticulocyte development [5]. Furthermore, synergistic interaction of *Plasmodium vivax* reticulocyte binding protein 2b (PvRBP2b) and *P. vivax* Duffy-binding protein (PvDBP) with reticulocyte-tropic transferrin receptor 1 (TfR/CD71) and Duffy receptor antigen for chemokines (DARC), respectively, provide compelling evidence of erythroblast and reticulocyte infection in haemopoietic tissue niches including bone marrow [4]. The parasite would thus appear to have almost no opportunity to reproduce in peripheral blood but, would have almost limitless access to cells bearing that receptor in the extravascular spaces of haemopoietic tissues. Indeed, recent work with *P. vivax* in splenectomized rhesus macaques demonstrated those spaces of bone marrow as the dominant compartment of that infection [7]. *Plasmodium falciparum* has also been shown to interact with bone marrow; employing it as a site of maturation and for evasion of the host immune response [8–13]. For initiation of active *P. falciparum merozoites entry*, the erythrocyte binding-like (EBL) and reticulocyte binding-like (Rh) protein families are responsible for binding to specific erythrocyte receptors [14]. In fact, higher prevalence of immature *P. falciparum* immature gametocytes in bone marrow than in peripheral blood has been demonstrated using quantitative polymerase chain reaction [15]. The anaemia that often develops with acute malaria involves bone marrow suppression and ineffective erythropoiesis [16–19].

Samples of bone marrow are obtained by an invasive procedure and collected when clinically indicated by suspicion of specific illnesses, or at autopsy. The presence of malarial parasites in the bone marrow was first reported in 1894 in malarial fevers occurring during summer and autumn [20]. Later, bone marrow aspiration was described as an accessory and useful tool in the diagnosis of malaria especially in cases where strong suspicion of malaria could not be confirmed on routine peripheral smears [9–11]. Peripheral smear examination under light microscopy remains the gold standard of malaria diagnosis and consistently negative findings in a patient are considered definitive. Point-of-care rapid diagnostic tests (RDTs) on peripheral blood are also widely used. However, malaria in endemic zones is often dominated by sub-patent and typically asymptomatic infections [21]. The finding of *Plasmodium* in samples of bone marrow in endemic settings may thus occur with negative findings from peripheral blood [22–24].

Currently, bone marrow trephine biopsy and aspiration are routinely done for the workup of various haematological, infiltrative and storage abnormalities [25, 26]. In general, plasmodial infection of bone marrow is usually an incidental finding to those investigations. It is not clear if undiagnosed malaria explained the illnesses prompting bone marrow examination, or if it was clinically silent and of no direct relationship to the presenting illness. The findings on bone marrow of patients with malaria range from normocellular, normoblastic erythropoiesis to hypercellular, dyserythropoietic erythropoiesis [27–29]. The current study adds to that scant body of evidence by reviewing the finding plasmodial infection incidental to bone marrow examination at teaching hospital in Karachi, Pakistan.

## Methods

A retrospective record review of all malaria parasite-positive bone marrow specimens examined at Aga Khan University Hospital (AKUH), a large tertiary care hospital in Karachi, Pakistan, from the years 2007 to 2015 was conducted. Endemic malarial transmission occurs seasonally in and around Karachi, and across much of Pakistan [30].

Bone marrow aspiration was either done at Aga Khan University, Main Campus or submitted for review at one of the collection points across Pakistan. A complete blood cell count and peripheral blood smear was also routinely done at the time of bone marrow aspiration. In all cases, bone marrow aspirate and trephine biopsy were collected from the posterior superior iliac spine. Bone marrow aspirates were collected as per International Council for Standardization in Haematology (ICSH) guidelines. Ten to 20 ml plastic syringe, without anticoagulant, was used to draw out the aspirate. The first 0.5ml were used to produce smears at the bedside while the remainder was put in a tube containing a proportional amount of di-potassium ethylenediaminetetraacetic acid (EDTA) to prevent sample coagulation. Bone marrow slides were prepared immediately after aspiration. The slides were stained with Leishman stain for microscopy.

Core biopsies of bone were taken using a Jamshidi needle. Whenever possible, a 2 cm long core was obtained in each case. Specimens were fixed and transported in 10 % buffered formalin. After decalcification in 10 % formic acid, paraffin-wax embedding was done. Sections, 4-µm‐thick, were cut by experienced histotechnologists using standard equipment and used for haematoxylin and eosin staining.

Patients included in the study were either already diagnosed with malaria and their bone marrow was examined for work-up of another condition, or the finding of malarial pigment/parasites was incidentally discovered in the bone marrow sample.

A peripheral blood smear was considered positive when any plasmodial life cycle stage was identified. In addition, bone marrow aspirate was considered positive for malarial parasite when any stage of plasmodial life cycle or haemozoin was identified.

Following exemption from the ethics review committee (3672-Pat-ERC-15), anonymized data was collected from past records and analysed using Statistical Package for the Social Sciences (IBM SPSS) version 19. The data reviewed for each patient included: (a) patient demographics, (b) relevant, significant findings on history and physical examination, (c) a complete blood count and blood peripheral film of a blood sample taken at the time of bone marrow sampling and (d) final report of bone marrow aspirate and/or trephine biopsy done on bone marrow samples.

Frequencies and percentages were calculated for categorical variables and mean and standard deviation for continuous variables. For statistical analysis, Fisher’s exact test was used for categorical variables whereas Mann-Witney U was employed for continuous data.

## Results

**Fig. 1:**
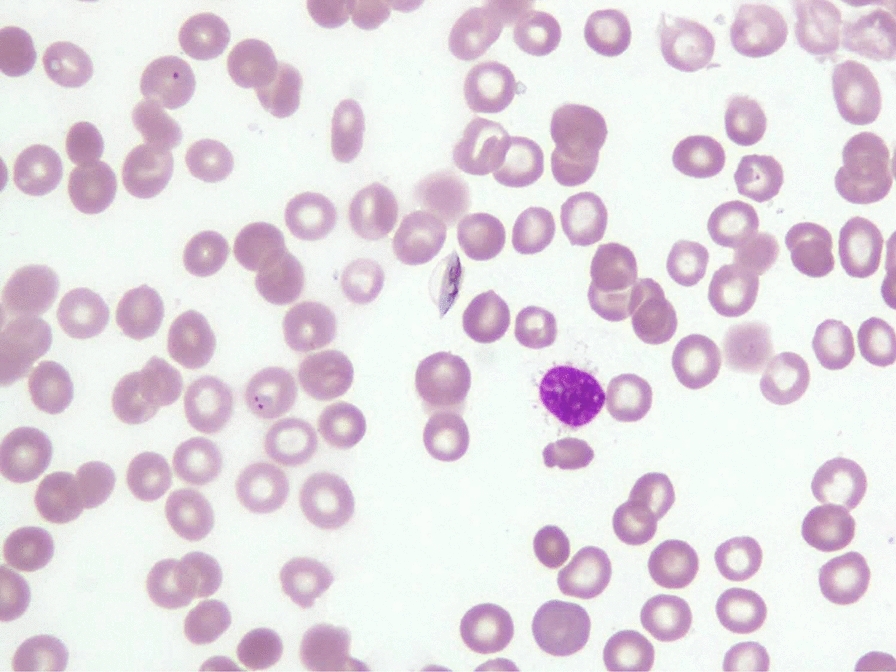
Malaria parasite *Plasmodium falciparum*, trophozoites and gamete within a sample aspirated from the bone marrow

**Fig. 2:**
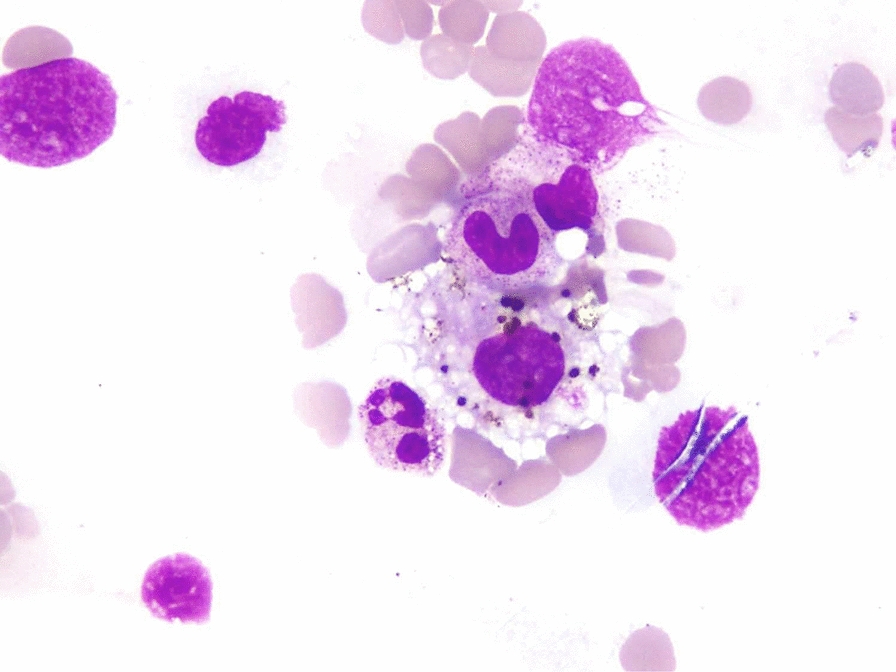
Bone marrow aspirate showing haemozoin with in a histiocyte

**Fig. 3:**
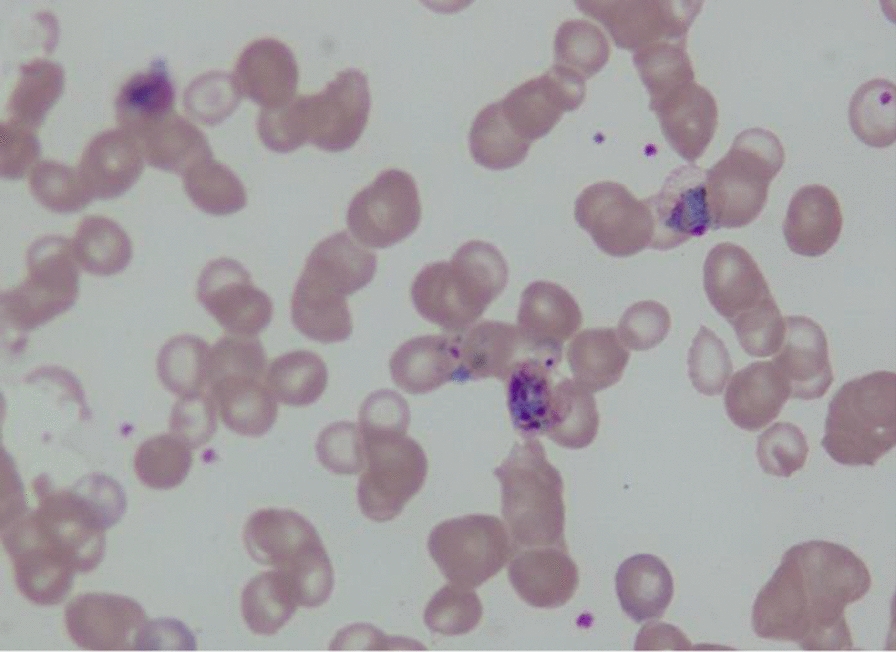
Malaria parasite *Plasmodium vivax*, trophozoites, gametes and schizont in various stages of development within as sample aspirated from the bone marrow

A total of 25,867 bone marrow samples were received in laboratory during the study period. Thirty-five (0.13 %) patients were reported as positive for malaria parasites. Six of 35 samples were received from outside Karachi. The median patient age was 22 years (range 1–75), comprising of 60 % (n = 21) male patients. Fever and cytopenias were the two most common indications for bone marrow examination, accounting for approximately two thirds of cases (Table 1). Hepato/splenomegaly was the next most common indication. All the patients were symptomatic and the most common symptoms reported were fever and fatigue (Table 2). *Plasmodium falciparum* was identified in 22 patients, whereas, 12 patients had *P. vivax* (Figs. 1, 2 and 3), and one patient had a mixed species infection. Table 3 lists the various stages of *Plasmodium* identified in blood and bone marrow. Four patients were positive by marrow examination but not peripheral blood smear. Three of these had *P. falciparum* infection and a fourth patient had a mixed infection. In another patient with *P. vivax* in peripheral blood, haemozoin was the only evidence of plasmodial infection of the bone marrow (Table 3). Of all the complete blood count parameters, haemoglobin and haematocrit were significantly lower in patients with *P. falciparum* (P = .003 and .001 respectively). This indicates more severe anaemia associated with this sub-species (Table 4). The higher mean corpuscular haemoglobin with borderline P value of 0.049 in *P. falciparum* cohort cannot be explained.

**Table 1:** Indications for bone marrow examination in cases later found to be positive for malaria

Indication of bone marrow biopsy	N (%)
Fever	13 (37.1)
Cytopenias	
Anaemia	1 (2.8)
Thrombocytopenia	3 (8.6)
Bi or pancytopenia	8 (22.8)
Hepato/splenomegaly	7 (20)
Staging Hodgkin’s lymphoma	1 (2.8)
Staging of Neuroblastoma	1 (2.8)
For remission status of Leukaemia	1 (2.8)

**Table 2:** Clinical features of the case series

Symptoms and signs reported	n (%)
Fever	22 (62.85 %)
Pallor	17 (48.57 %)
Fatigue	18 (51.42 %)
Weight loss	14 (40 %)

**Table 3:** Various *Plasmodium* stages and haemozoin detected in peripheral blood (PB) and Bone marrow (BM)

Species	Trophozoites on PB	Gametocytes on PB	Schizonts on PB	Trophozoites on BM	Gametocytes on BM	Schizonts on BM	Hemozoin in BM
*P. vivax* (n = 11)	10 (83.3)^a^	10 (83.3)^a^	2 (16.7) ^a^	9 (75.0)	9 (75.0)	2 (16.7)	4 (33.3)
*P. falciparum* (n = 22)	11 (50.0)	11 (50.0)	–	12 (54.5)	15 (68.2)	–	11 (50.0)
Mixed (n = 1)	1 (100.0)	1 (100.0)	–	1 (100.0)	1 (100.0)	–	1 (100.0)

^a^For one patient a peripheral blood sample was not available

**Table 4:** Complete blood count parameters in patients with *P. vivax* and *P. falciparum*

Haematological parameters	*Plasmodium vivax*	*Plasmodium falciparum*	P-value
Hb (g/dl)	9.77 (± 1.44)	7.73 (± 1.72)	0.003
Haematocrit	30.17 (± 4.73)	23.17 (± 4.81)	0.001
Platelet Count	74.45 (± 74.82)	81.5 (± 67.49)	0.801
WBC	5.9 (± 2.84)	5.18 (± 3.22)	0.549
ANC	3 (± 2.19)	2.05 (± 0.75)	0.18
MCV	80.9 (± 4.8)	85.3 (± 10.6)	0.206
MCH	26.26 (± 2.11)	28.29 (± 3.23)	0.049

## Discussion

In Pakistan, the two most prevalent causes of malaria are *P. vivax* and *P. falciparum*, constituting 78 and 21 % of cases, respectively [1]. In contrast, most of the infections in our case series were *P. falciparum* (63 %). The authors do not believe that the bone marrow samples were taken on suspicion of or for confirmation of malaria. The diagnosis may be viewed as an incidental finding in bone marrow examination undertaken for various other indications (Table 1). However, since most samples were received from outside our centre, the result of microscopic examination of peripheral blood films routinely done prior to ordering the bone marrow procedure is not known. Ideally, bone marrow examination would not have been performed in cases where peripheral blood smear was positive for malaria parasite. Only 4 of our 35 patients had no evidence of *Plasmodium* on the peripheral films. Whether the parasites were missed on the initial peripheral smears, or were absent in early peripheral smears cannot be confirmed.

The primary cause of illness among our 35 patients appeared to be consistent with chronic low-grade plasmodial infection. Most of them were markedly anaemic (mean of 8.6 g/dL) and they recovered with anti-malarial therapy. So-called “asymptomatic malaria” having chronic, low-grade parasitaemias are indeed associated with states of anaemia [31]. Pathologists examining bone marrow should be alert to the possibility of malaria as the cause of illness, prompting appropriate examination.

It may be likely that most cases of malaria in catchment area were indeed detected prior to initiating the bone marrow sample, as suggested by the low frequency of malaria (0.13 %) among the 25,867 bone marrow examinations we evaluated. The number of bone marrow examinations averted by an early diagnosis of malaria on peripheral blood smear cannot be counted, but it may be considerably larger than the cases described here. At least in the small sample of bone marrow samples found positive for malaria, the even rarer numbers lacking parasites on peripheral blood smear suggests that infection of bone marrow without parasitaemia may be the exception. A systematic review of infections among blood donors during 2010 to 2020 in Pakistan found 0.11 % positive for malaria [32]. It cannot be known if invasion of blood by the *Plasmodium* is usually attended by infection bone marrow, the similarities of those rates of infection of blood and bone marrow suggests that may be the case.

It is well known that bone marrow aspirates may be contaminated to a variable extent with cells from peripheral blood. Accurate discriminant analysis enumerating plasma cells, CD34 + cells and CD10 + granulocytes by flow cytometry as described in literature was not possible in current study [33]. The authors are nonetheless confident the aspirates were indeed dominated by elements of bone marrow (bone spicules, plasma cells, and megakaryocytes were present) and consider the *Plasmodium* seen therein as very probably residing in that tissue rather than being incidentally captured at biopsy.

## Conclusions

This case series of 35 patients found to have malaria presented with illnesses that prompted bone marrow examinations, and they represented a small minority of patients undergoing that procedure. Infection of bone marrow may be a common feature of malaria, be it acute or chronic in character.

## Data Availability

The datasets used and analysed during the current study are available from the corresponding author on reasonable request. Most of data analysed during this study are presented in this published article.
